# Supplementary material for: Global siRNA Screen Reveals Critical Human Host Factors of SARS-CoV-2 Multicycle Replication
Source: bioRxiv. 2024 Jul 10:2024.07.10.602835. Preprint. [Version 1] doi: 10.1101/2024.07.10.602835 (PMC11257544; doi:10.1101/2024.07.10.602835)

bioRxiv preprint doi: https://doi.org/10.1101/2024.07.10.602835; this version posted July 10, 2024. The copyright holder for this preprint (which was not certified by peer review) is the author/funder, who has granted bioRxiv a license to display the preprint in perpetuity. It is made available under aCC-BY 4.0 International license.

SUPPLEMENTAL FIGURE LEGENDS

Figure S1 – Genome-wide siRNA screen identifies host factors involved in SARS-CoV-2 replication

(A) Dot plot shows average SARS-CoV-2 infectivity Z-score values from the genome-wide siRNA screen. Controls are shown (non-targeting scrambled siRNA, negative; siACE2 and siTMPRSS2, positive). (B) Correlation plots of Z-score values for genome-wide siRNA screens using Caco-2 cells infected with SARS-CoV-2. R = Pearson correlation coefficient between screens.

Figure S2 – Expression of the identified host factors in SARS-CoV-2 target cells

(A) Heatmap shows percentage of detectable levels of expression of a given factor in the indicated cell type[73]. % expression >1 was considered a detectable level. (B-G) Zoom-in insets from selected biological processes are indicated with an asterisk * in the hierarchy. The nodes indicate host factors and their color matches the dataset where they were identified. Edges indicate interactions from STRING database. Grey nodes indicate SARS-CoV-2 proteins.

Figure S3 – Mapping of host factors into SARS-CoV-2 infectious cycle

(A) Caco-2 cells subjected to siRNA-mediated knockdown of the indicated host factors were infected with SARS-CoV-2 pseudotyped VSV luciferase virus (VSV-S) or VSV luciferase virus expressing its natural glycoprotein (VSV-G) for 18h prior to measurement of luciferase signal. Data represent mean from one representative experiment in duplicate (n=2). (B,C) Binding of spike protein and RBD to perlecan. Surface plasmon resonance (SPR) was used to evaluate spike binding to perlecan. This experiment was repeated twice.

Figure S4 – Pharmacological inhibition of BIRC2 reduces SARS-CoV-2 replication *in vitro* and *in vivo*

bioRxiv preprint doi: https://doi.org/10.1101/2024.07.10.602835; this version posted July 10, 2024. The copyright holder for this preprint (which was not certified by peer review) is the author/funder, who has granted bioRxiv a license to display the preprint in perpetuity. It is made available under aCC-BY 4.0 International license.

(A) Cells were treated with AZD5582 at the indicated concentrations. 24 hours post-treatment, the cell lysates were analyzed by Western blotting for p100/p52 protein. A representative immunoblot presented here demonstrate that AZD5582 treatment induces the cleavage of p100. (B) Layout of mice experiments using SARS-CoV-2 B.1.1.7 (Alpha) infection. Effect of AZD5582 on SARS-CoV-2 replication in survival (C) and body weight (D) were recorded for 14 days post-infection. Virus titer as measured in the lungs of infected mice by plaque assay (E) were performed on 3dpi. Tissue sampling was done at 72hpi. One-way ANOVA when compared with the vehicle control group. *P<0.05, ****P<0.001.

Figure S1. Genome-wide screen identifies host factors involved in SARS-CoV-2 replication

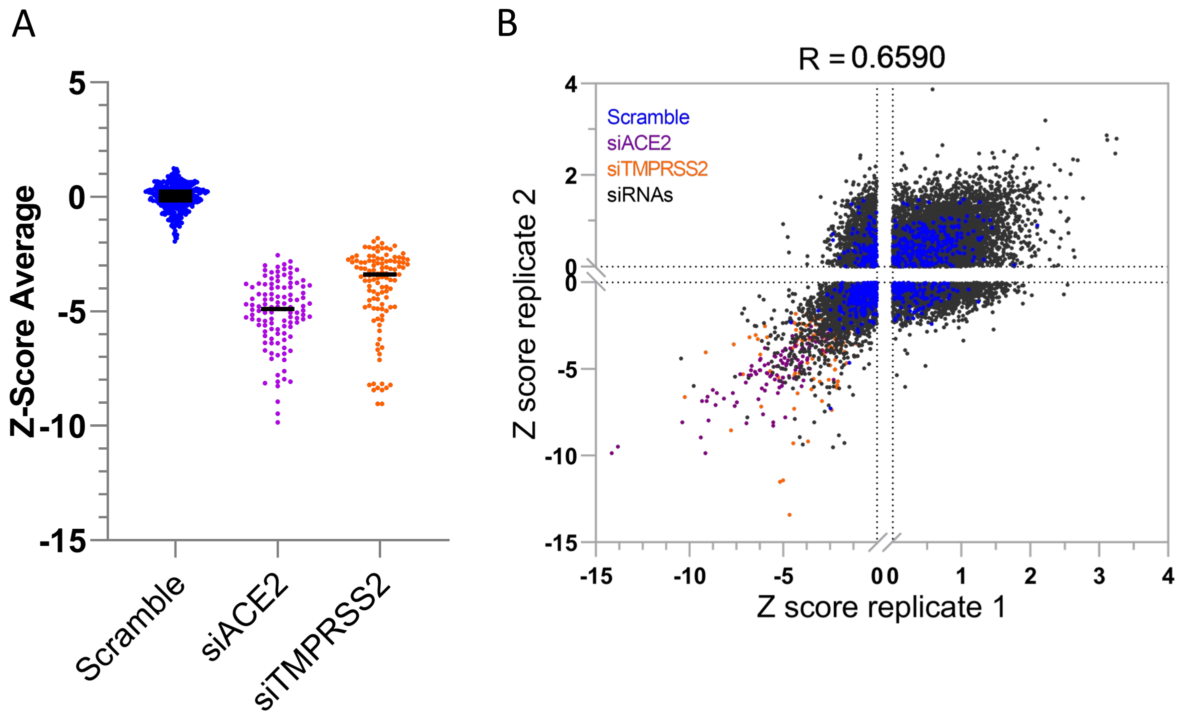

Figure S2. Expression of the identified host factors in SARS-CoV-2 target cells

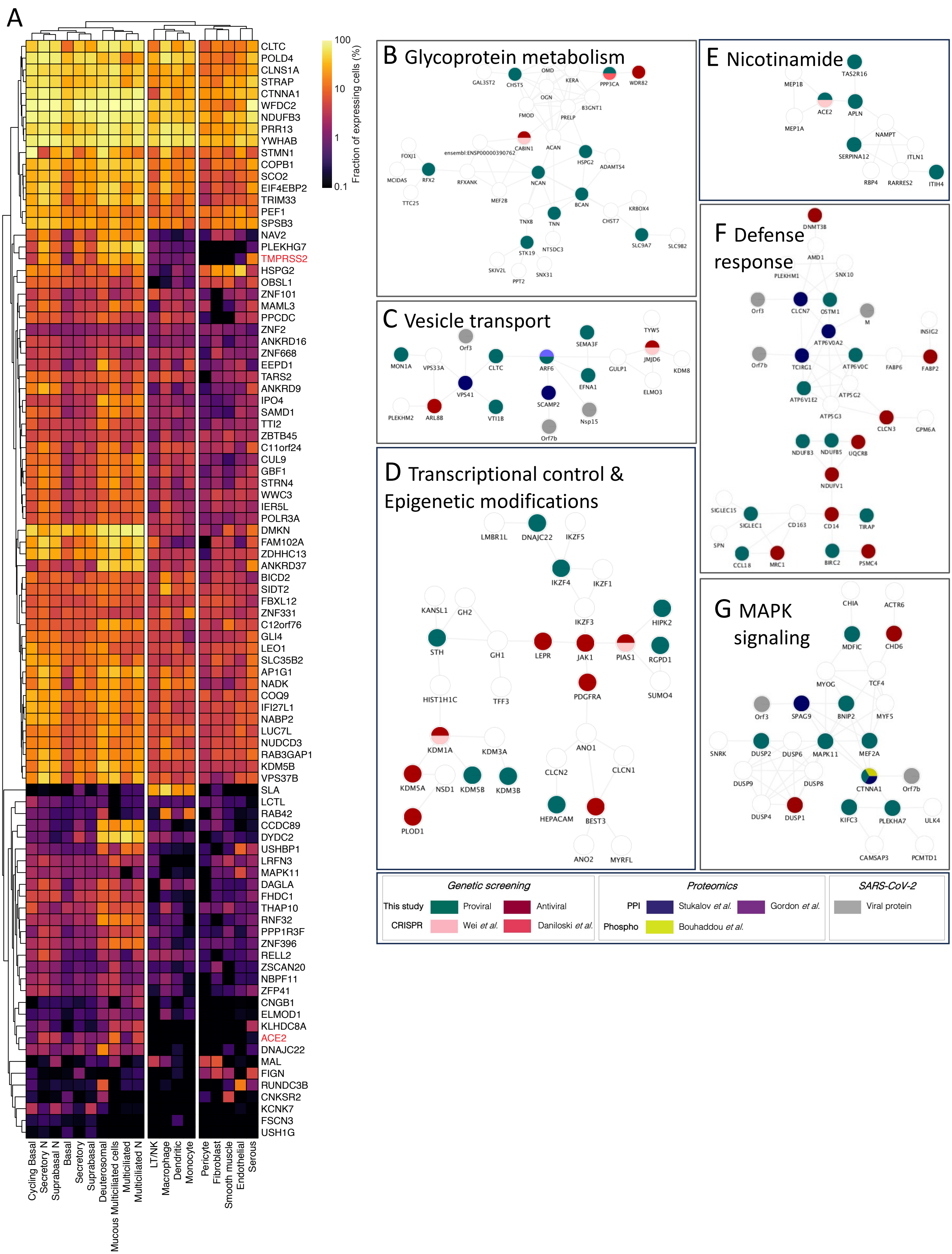

Figure S3. Mapping of host factors into SARS-CoV-2 infectious cycle

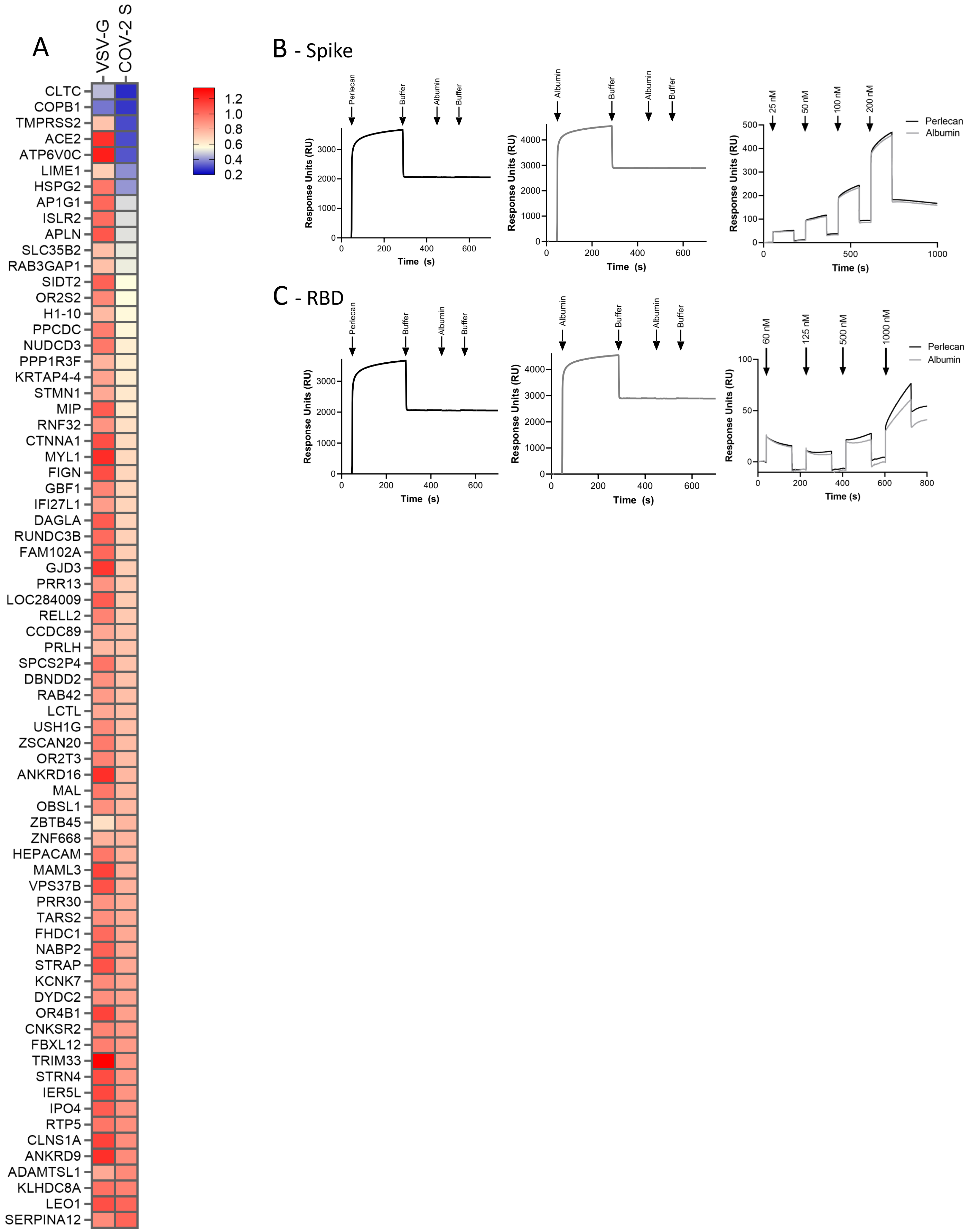

Figure S4. Pharmacological inhibition of BIRC2 reduces SARS-CoV-2 replication *in vitro* and *in vivo*

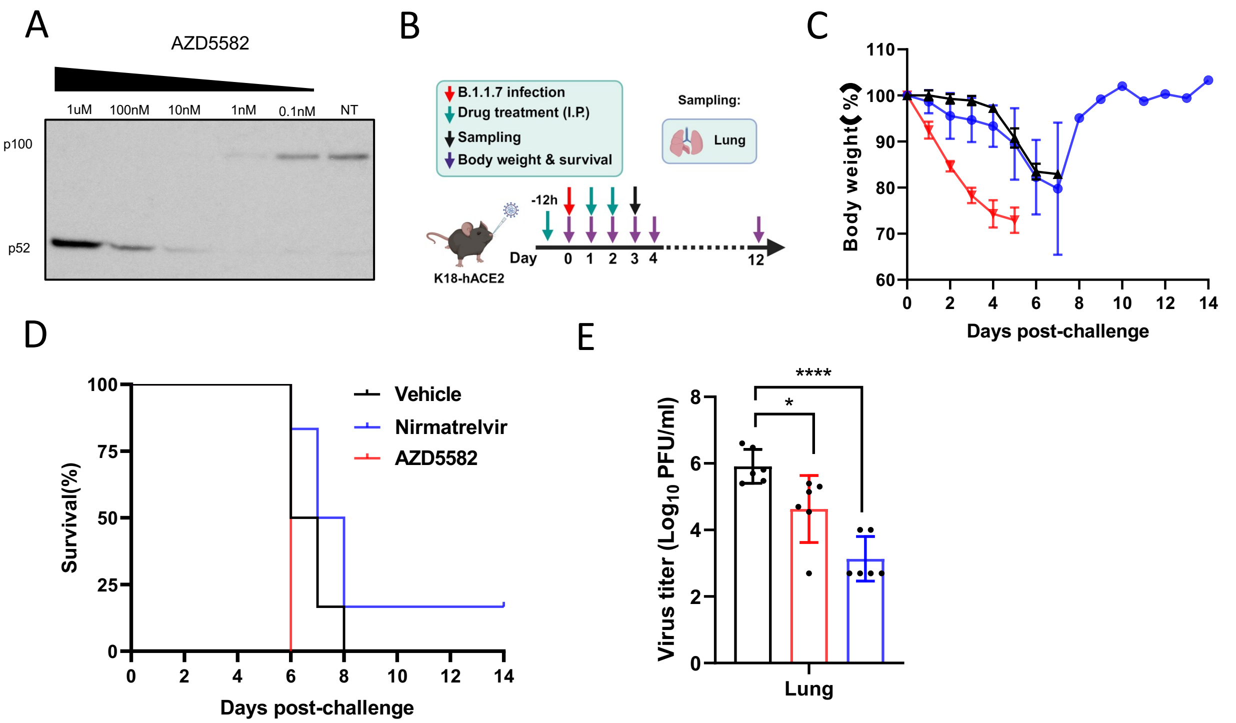

Supplement: Supplement 1 [file NIHPP2024.07.10.602835v1-supplement-1.pdf]
